# 

**Published:** 1897-11

**Authors:** 


*Fifty-Third Year.*

VOL. LIII.

WHOLE NUMBER,

DCXII.

NOVEMBER, 1897.

New Series.
VOL. XXXVII.
No. 4.

LIBRARY
SURGEON GENERAL'S OFF
NOV.-1--1897

### BUFFALO MEDICAL JOURNAL

Established 1845 by AUSTIN FLINT, M. D.

EDITORS:

THOS. LOTHROP, M. D. WM. WARREN POTTER, M. D.

ASSOCIATE EDITORS:

WILLIAM C. KRAUSS, M. D. JAMES WRIGHT PUTNAM, M. D.
JOHN A. MILLER, PH. D. JOHN PARMENTER, M. D.
ERNEST WENDE, M. D. ALVIN A. HUBBELL, M. D.

SCRIPTA FERUNT ANNOS

EUROPEAN AGENCY, J. B. BAILLIÉRE & FILS, 19 Rue Hautefeuille, Paris
*Subscription, if paid in advance, $2.00; otherwise, $2.50. Foreign subscription, $2.50.*
Copyright 1897, by WILLIAM WARREN POTTER, M. D.
Entered at the Post Office at Buffalo, N. Y., as second-class mail matter.

A. T. BROWN PRINTING HOUSE, BUFFALO, N. Y.

Table of Contents on Page V.

